# Longitudinal analysis of neuromuscular adaptation using entropy and PCA during motor learning

**DOI:** 10.1186/s40101-026-00432-y

**Published:** 2026-05-15

**Authors:** Megumi Shimura, Yoshihiro Shimomura

**Affiliations:** 1 Graduate School of Science and Engineering, Chiba University, 1-33 Yayoicho, Inage-ku, Chiba-shi, Chiba, 263-8522 Japan; 2 Design Research Institute, Chiba University, 1-33 Yayoicho, Inage-ku, Chiba-shi, Chiba, 263-8522 Japan

**Keywords:** Motor learning, Surface electromyography, Entropy, Principal component analysis, Longitudinal analysis

## Abstract

**Background:**

Motor learning can yield clear behavioral gains even when EMG-derived indices show subtle, heterogeneous, or non-monotonic change. We aimed to describe longitudinal trajectories of performance and multi-channel EMG summaries during extended practice of a tempo-constrained finger sequencing task under stable constraints.

**Methods:**

Eleven participants practiced a fixed sequence for 30 sessions. Timing accuracy was quantified as mean absolute timing error. Forearm/hand EMG was normalized to a reference voluntary contraction (%RVC). We computed EMG entropy as a summary of the spatial dispersion of activation, summarized early-to-late changes in normalized amplitude (Δ%RVC), and used a pooled (common) PCA framework to define a dataset-level dominant covariation axis and track session-related changes in PC1 scores (projections) along this fixed axis. Longitudinal trends were examined using linear mixed-effects models.

**Results:**

Timing error decreased with practice in a pattern consistent with a negatively accelerated (quadratic) trajectory. Group-level entropy did not show a uniform monotonic trend, and individual entropy trajectories varied substantially. Δ%RVC changes were generally small and non-uniform across muscles and participants. In the pooled PCA framework, PC1 scores exhibited a modest session-related drift, suggesting gradual shifts in activation patterns along a common covariation dimension.

**Conclusion:**

Under fixed task constraints, behavioral improvement may coexist with heterogeneous EMG entropy trajectories and small, non-uniform changes in normalized amplitude. Multi-channel summaries can help characterize individualized routes to skill acquisition, whereas mechanistic interpretation will likely require additional measures (e.g., kinematics/kinetics) and/or explicit manipulations of task constraints.

**Supplementary Information:**

The online version contains supplementary material available at 10.1186/s40101-026-00432-y.

## Background

Motor learning is commonly defined as a set of processes associated with practice or experience that leads to relatively permanent changes in the capability for skilled performance [1]. Beyond behavioral improvements in accuracy and consistency, learning is accompanied by neuromuscular adaptations observable in electromyographic (EMG) measures and motor unit behavior. Prior studies have reported practice-related changes in EMG amplitude and timing, modifications of agonist–antagonist co-contraction, and reorganization of motor unit discharge behavior, suggesting that performance improvements may coincide with systematic changes in muscle recruitment and coordination, although such changes are not necessarily uniform across individuals or tasks [2–5].

A central feature of human movement is the high dimensionality of the musculoskeletal system, particularly in the forearm–hand complex. The human hand enables a wide repertoire of grasping and manipulation, supported by thumb opposability and anatomical features that facilitate both power and precision grips [6, 7]. The hand contains many joints and degrees of freedom, while numerous intrinsic and extrinsic muscles contribute to overlapping mechanical actions; in addition, tendinous and soft-tissue couplings impose structural constraints such as interdependence among digits [8, 9]. At a broader level, the motor system contains more degrees of freedom than are minimally required to achieve typical task goals, yielding multiple feasible solutions for the same task outcome—an issue historically framed as the “degrees-of-freedom” or motor redundancy problem [10]. Consistent with the “motor abundance” perspective, this multiplicity of solutions implies that distinct muscle-use patterns can realize similar performance, making individuality in neuromuscular organization a plausible and expected feature of learning [11].

Despite well-documented group-level trends in neuromuscular adaptation, it remains unclear how muscle activation patterns evolve dynamically within individuals across practice. Many studies emphasize endpoint contrasts (e.g., novice–expert comparisons or pre–post designs), which can overlook nonlinear time courses and within-person reorganization processes. Increasingly, motor learning is recognized as individualized: substantial inter-individual differences exist in learning rates, attainable performance levels, and the paths by which improvement is achieved [12]. Motor learning has also been framed as a search process within a perceptual–motor workspace, in which individuals may follow different routes to achieve stable task solutions under shared constraints [13, 14]. Such variability is not merely “noise” but may reflect stable differences in sensory and neural characteristics related to learning. For example, individual differences in motor learning have been linked to structural and functional brain measures [15] and to proprioceptive variability predicting the extent of implicit sensorimotor adaptation [16]. These perspectives motivate longitudinal approaches that preserve individual trajectories rather than relying solely on group averages [12, 17].

For high-dimensional systems such as the forearm and hand, adaptation is likely expressed not only as changes in overall activation level but also as reorganization of coordination structure, that is, functional patterns of relationships among muscles that support task achievement. In the present study, coordination is defined as a functional integration pattern among multiple muscles, including how roles are distributed and how muscles covary during task execution [18]. A prominent account is that the central nervous system may recruit muscles in groups or modules (often discussed in the context of muscle synergies), which can reduce effective control dimensionality and enable flexible generation of diverse movements through combinations of a limited set of units [19, 20]. Empirically, coordination patterns can change with skill and context: co-contraction can stabilize movement at the cost of increased activation, while other strategies involve redistributing activation or alternating activity across synergists to sustain performance [21–24]. These considerations suggest that learning may be characterized by reweighting within existing coordination patterns and, in some cases, restructuring of coordination structure, potentially via multiple acceptable solutions rather than a single canonical pattern.

Accordingly, this study characterizes learning-related neuromuscular adaptation from two complementary perspectives: (1) the spatial distribution of muscle activation across the forearm and (2) the covariation structure among muscles. To quantify distributional characteristics, we use an entropy-based index grounded in information theory, where entropy summarizes the dispersion (evenness) of a distribution rather than prescribing a priori which spatial pattern is “optimal” [25, 26]. Entropy measures have been applied to physiological signals, including EMG, as complexity-related descriptors of state transitions such as fatigue [27, 28], and information-theoretic features have also been used for EMG pattern representation [29]. In the present context, we operationalize entropy specifically as a summary index of spatial dispersion of activation across simultaneously recorded forearm muscles, enabling a compact description of whether activity is concentrated in a subset of muscles or distributed more broadly. Importantly, the magnitude of entropy is not treated as intrinsically “good” or “bad”; rather, it is interpreted as a descriptive marker of distributional state, and its meaning depends on task constraints and accompanying measures [30].

To characterize coordination structure, we apply principal component analysis (PCA) to multi-muscle EMG to summarize dominant covariation patterns and to identify a dominant covariation pattern (PC1 loading) from pooled multi-muscle %RVC vectors and track longitudinal change in the expression of this pattern via PC1 scores. PCA has been used to represent multi-muscle activation patterns in locomotion and other tasks and to compare coordination under different conditions and states [31–34]. Here, PCA outputs are treated as descriptive indices of coordination structure (i.e., how muscles co-vary and how those relationships shift), rather than as direct evidence of efficiency.

To examine learning-related reorganization of forearm muscle activity under stable constraints, we employed a fixed-sequence four-key press task simulating piano performance. Participants executed a repeating “score” in which visual note stimuli descended in four lanes at a constant tempo, and they were instructed to press the corresponding key as each note aligned with a judgment line. The task provided immediate categorical feedback on timing accuracy and auditory feedback for correct presses, supporting incremental improvement across repeated trials. Because the sequence, tempo, key–lane mapping, and feedback rules were held constant across sessions, the task enables longitudinal comparison of neuromuscular patterning under consistent demands, while still requiring temporally precise finger actions and coordination across multiple digits.

The protocol was explicitly designed to capture individual trajectories by repeating practice across 30 sessions with standardized structure and rest periods to mitigate fatigue effects, complemented by a brief key-tapping check before each session. By tracking performance together with entropy-based dispersion and PCA-based coordination structure over time, the analysis can test whether individuals show gradual, abrupt, or non-monotonic changes in neuromuscular organization across practice.

The purpose of this study is to characterize individual learning trajectories during short-term practice of a fixed-sequence four-key press task by longitudinally tracking task performance together with (i) entropy-based spatial dispersion of forearm muscle activation and (ii) PCA-based coordination structure among muscles. By integrating distribution- and structure-level descriptions with a trajectory-focused analysis, we aim to document individual differences in learning-related neuromuscular adaptation beyond what is captured by performance alone.

## Methods

### Participants

Eleven healthy adults (6 males, 5 females; mean age = 26.6 ± 2.9 years) participated in the study. Handedness was assessed using the Edinburgh Handedness Inventory, and all participants were confirmed to be right-handed. None of the participants reported a history of injury or neurological disorders affecting the dominant hand, and none reported injuries or conditions involving the dominant hand within the 6 months prior to the experiment.

To minimize the influence of prior musical expertise, participants were excluded if they had more than 1 year of piano practice beyond typical school education. Here, “piano practice” was defined as structured training such as private lessons, club activities, or sustained self-directed practice. All eligibility information was obtained by self-report.

This study was approved by the Ethics Committee of the Graduate School of Science and Engineering, Chiba University (Approval No. R6-29).

### Task

**Fig. 1:**
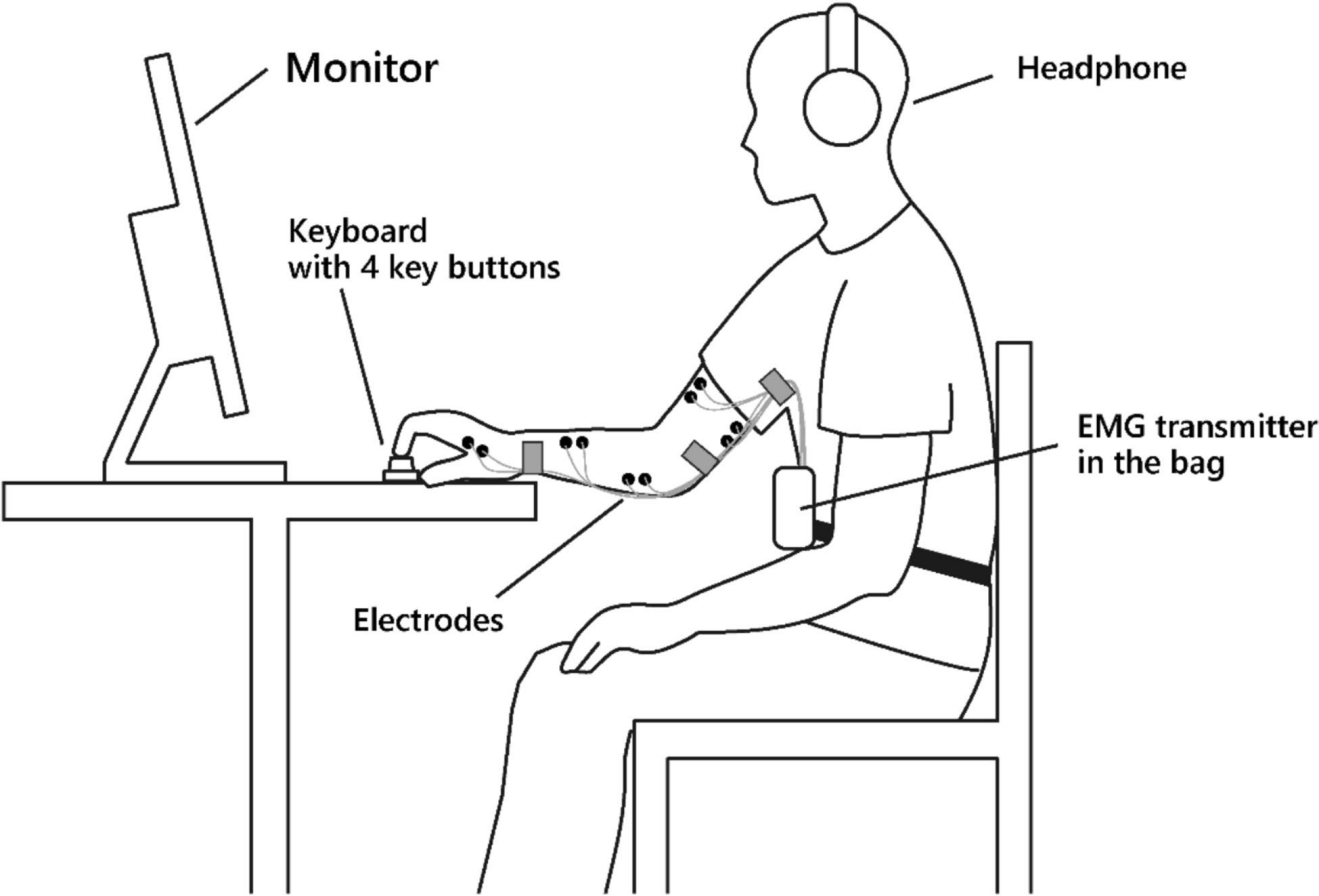
Illustration of the participant posture and experimental setup. Participants sat in front of a monitor with their right hand placed on a four-key keyboard. Visual stimuli were presented on the monitor, and participants pressed the keys using their index to little fingers. Auditory feedback associated with each key press was delivered via headphones. Surface electromyography (sEMG) electrodes were attached to the right upper limb, and electrode cables were bundled and secured along the right arm and trunk using surgical tape to minimize movement artifacts. The wireless EMG transmitter was placed in a small bag and fixed to the abdomen. Detailed electrode locations are shown in Fig. 5

Participants were seated in front of a desk, with the right hand placed on the desk and the left hand positioned comfortably on the lap or desk surface. The setup included a monitor (509.18 × 286.42 mm, VZ239HR, ASUS) and a custom keyboard with four key buttons (MK-04WH, ITPROTECH CO., LTD.) (Fig. 1). Participants wore headphones throughout the task and were instructed to keep the second to fifth fingers of the right hand on the keyboard. Screen resolution was 1280 × 720 pixels with a refresh rate of 60 Hz.

**Fig. 2:**
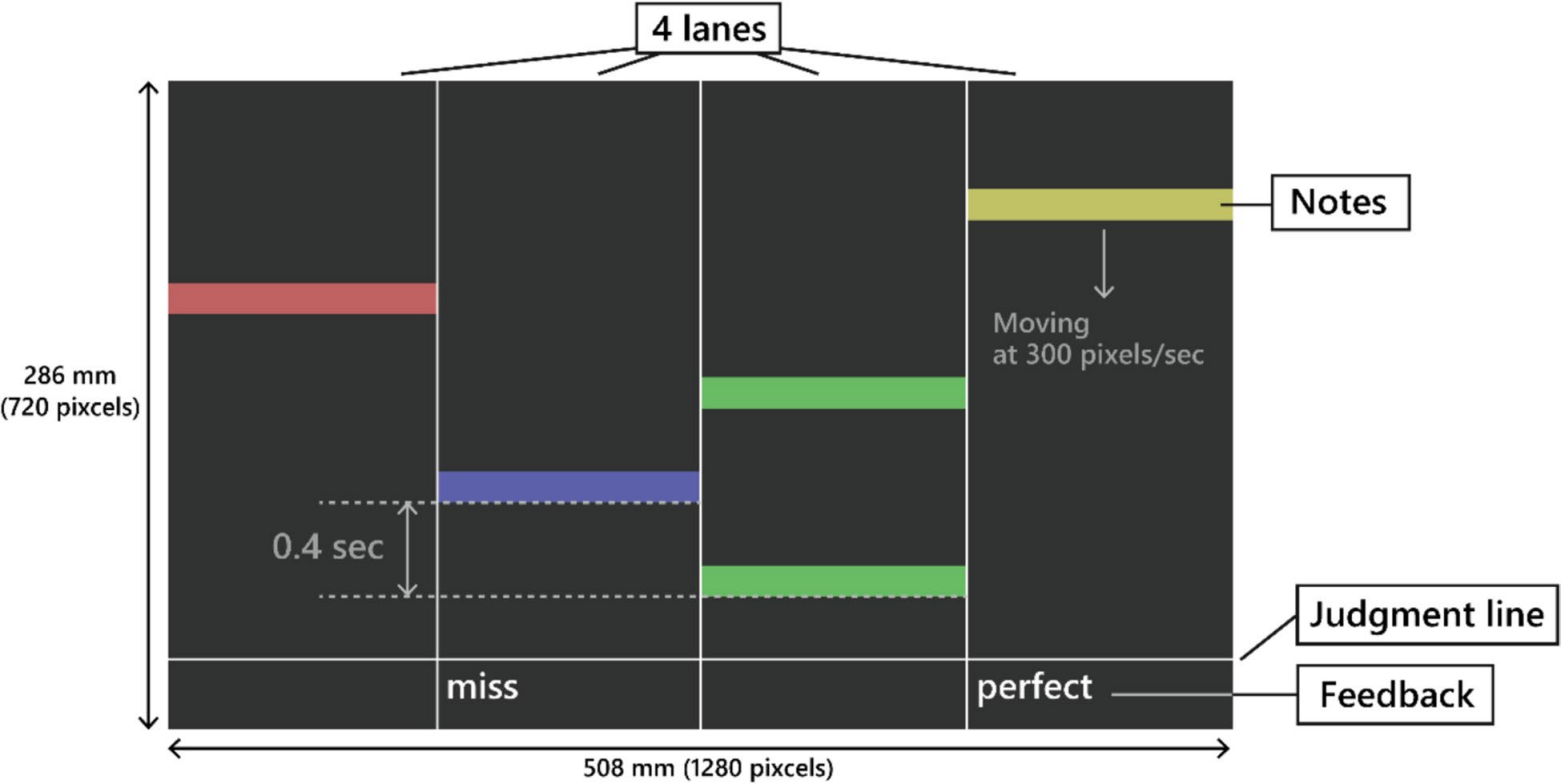
Four-lane key-press task design

The task was a temporally constrained finger sequencing task designed to examine longitudinal changes in timing accuracy and multi-finger coordination under fixed task constraints, rather than to assess musical skill. Visual stimuli (notes) descended from top to bottom in four lanes separated by white lines. Participants were instructed to press the corresponding key at the moment the bottom edge of each note aligned with a judgment line located 70 pixels from the bottom of the screen (Fig. 2). Notes appeared every 0.4 s and moved at 300 pixels/s.

**Fig. 3:**
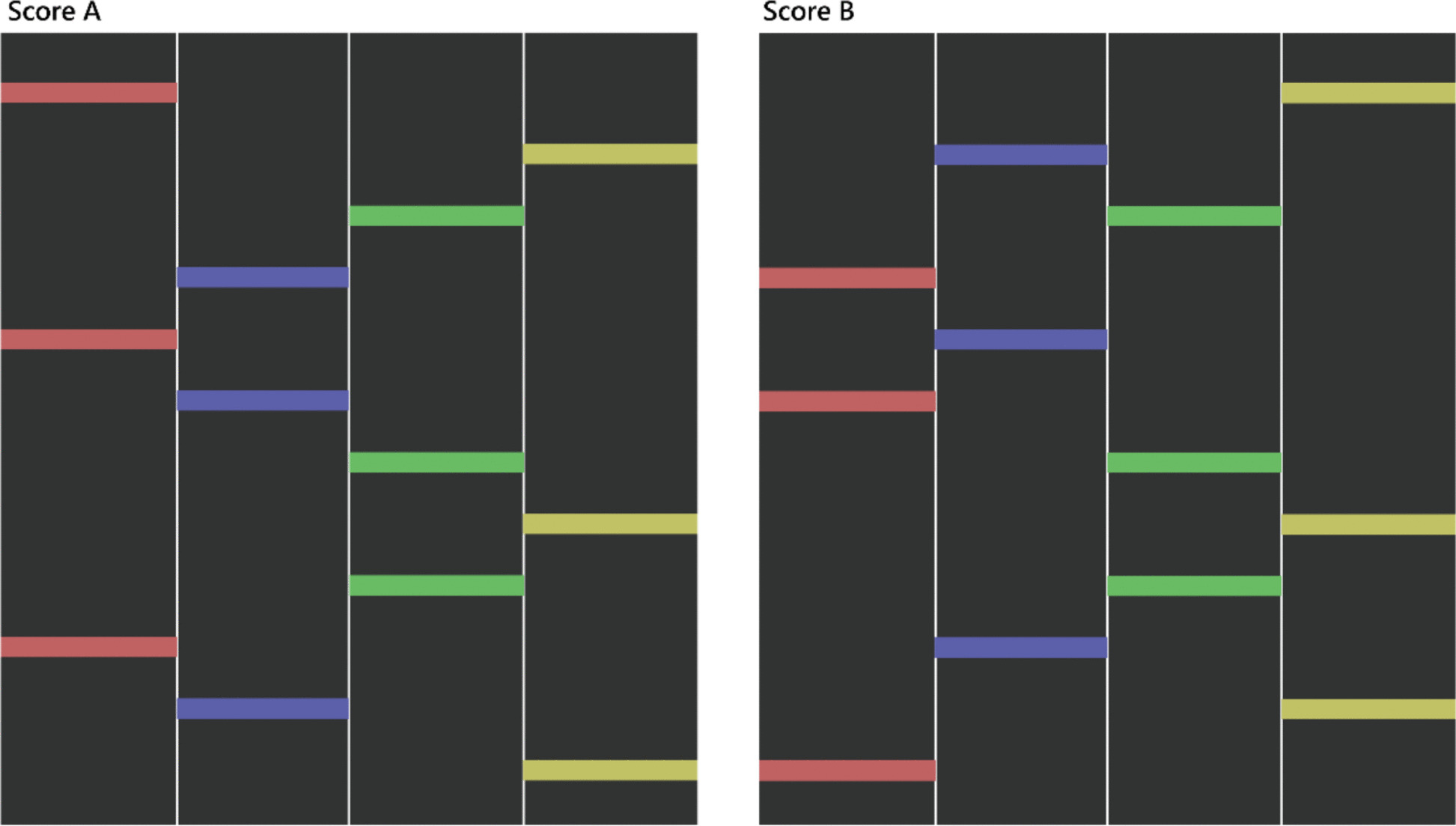
Predefined note sequences used in the finger sequencing task

The note order was not randomized. Instead, two predefined sequences (score A/B) were used, each consisting of 12 notes. In each sequence, each of the four fingers was required to press the corresponding key three times (equal finger-use frequency). The two scores are illustrated in Fig. 3. Two predefined scores were prepared to reduce potential sequence-specific influences, particularly finger transitions, under otherwise identical task constraints (tempo, note density, and equal finger-use frequency).

If the correct key was pressed while the note was within the response window defined as 200 pixels above and 70 pixels below the judgment line, the note disappeared, and the corresponding tone was presented through the headphones. Visual feedback indicating timing accuracy was displayed below the judgment line for 1 s: “miss” (> 0.25 s deviation), “good” (0.15–0.25 s), “great” (0.05–0.15 s), or “perfect” (< 0.05 s). Each task execution (one playback of the score) lasted approximately 6.8 s from the appearance of the first note to the disappearance of the last. After a 3-s pause, the same score was presented again.

**Fig. 4:**
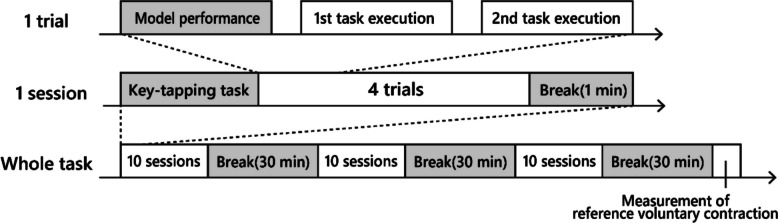
Task schedule and experimental structure

The overall task schedule and experimental structure are shown in Fig. 4. Each trial began with a model performance in which note stimuli were presented with synchronized auditory tones; participants could not interact with the keys during this period. The model performance was followed by two task executions. Each session included four trials (two trials per score). Sessions were separated by 1-min breaks, and a break of at least 30 min was given after every 10 sessions.

Before each session, a brief key-tapping task was administered to monitor fatigue: participants pressed the four keys as many times as possible within 3 s. If the total number of taps decreased by more than 10% compared to the first session, an additional rest period was provided.

The screen background was gray (RGB: 50, 50, 50); lane boundaries, judgment line, and feedback text were white (255, 255, 255). Notes were color-coded from left to right as red (200, 100, 100), blue (100, 100, 200), green (100, 200, 100), and yellow (200, 200, 100). The corresponding tones were 440 Hz, 660 Hz, 880 Hz, and 1320 Hz, respectively. The task was implemented in Python and executed using Microsoft Visual Studio.

In the present dataset, the two scores yielded comparable timing-error distributions and similar session-dependent trajectories; therefore, trials from score A and score B were combined and treated as repeated observations of the same task in all subsequent analyses. One session-level observation was missing due to an error in the recording log; therefore, the task-performance model was fitted to 329 observations (out of 330 possible). For each note, signed timing error was defined as $e={t}_{\mathrm{target}}-{t}_{\mathrm{press}}$. For each execution, we computed the mean absolute timing error across the 12 notes. If multiple presses occurred for the same key around a target, the press time closest to ${t}_{\mathrm{target}}$ was used. Presses occurring after the target but before the subsequent note event were still assigned to the intended note and included in the error calculation. Notes with no corresponding key press were treated as missing and excluded from the averaging for that execution.

### EMG recording and normalization

Surface electromyography (sEMG) was recorded from 10 muscles of the right upper limb using an EMG2-R amplifier and MP150 system (Biopac Systems, USA): biceps brachii, triceps brachii (long head), flexor carpi radialis, extensor carpi radialis, flexor carpi ulnaris, extensor carpi ulnaris, flexor digitorum superficialis, extensor digitorum, first dorsal interosseous, and abductor digiti minimi.

**Fig. 5:**
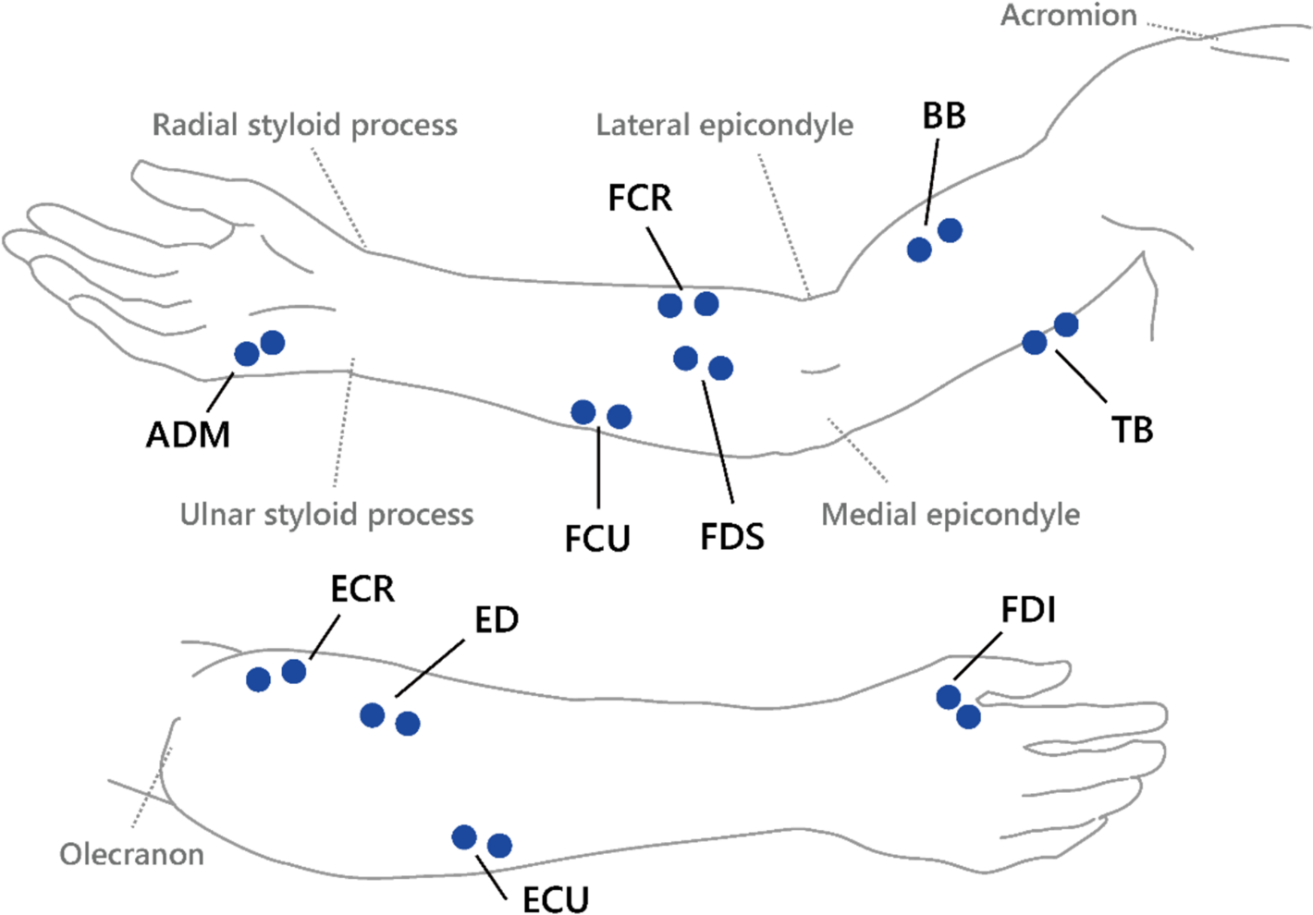
Electrode placement and anatomical landmarks

Before electrode placement, the skin was cleaned with cotton soaked in ethanol. Electrode locations were determined using anatomical landmarks and prior placement guidelines. Specifically: biceps brachii (BB, one-third distal on the line from acromion to cubital fossa), triceps brachii long head (TB, 50% on the line from posterior acromion to olecranon, two finger-widths medial), flexor carpi radialis (FCR, proximal one-third on the line between the FCR tendon at the wrist and the medial supracondylar area), extensor carpi radialis (ECR, most prominent region ~ 3 cm distal to the elbow), flexor carpi ulnaris (FCU, midpoint on the line between the ulnar styloid and medial epicondyle), extensor carpi ulnaris (ECU, midpoint on the line between the lateral epicondyle and ulnar styloid), flexor digitorum superficialis (FDS, mid-forearm between palmaris longus and FCU), extensor digitorum (ED, proximal one-third on the line between lateral epicondyle and the midpoint of the wrist), first dorsal interosseous (FDI, most prominent belly), and abductor digiti minimi (ADM, most prominent hypothenar belly) (Fig. 5). Interelectrode distance was 3 cm for upper arm and forearm muscles and 2 cm for intrinsic hand muscles. Ground electrodes were placed on the styloid process and the lateral epicondyle of the humerus. EMG leads were bundled and routed along the forearm. The wireless transmitter unit was secured in a pocket at the abdomen (Fig. 1).

Electrode–skin contact impedance was confirmed to be ≤ 5 kΩ. Signals were sampled at 2000 Hz. Representative raw EMG waveforms are provided in Fig. S1.

Signals were band-pass filtered with a Butterworth filter (20–450 Hz) to reduce motion artifacts and high-frequency noise. EMG amplitude was computed as a moving root mean square (RMS) using a 100-sample window (50 ms).

EMG amplitudes were normalized to the percentage of a reference voluntary contraction (%RVC) obtained during a maximal grip task measured with a hand dynamometer. Participants performed three 5-s maximal trials with 1-min rest intervals. RMS values were computed over the middle 3 s of each trial (using the same 100-sample RMS window), and the maximum RMS value across trials was used as the RVC. The grip task was conducted after completion of the motor-learning task, following a 30-min rest period.

### Entropy analysis

To quantify how widely muscle activation was distributed across the ten recorded forearm muscles, we computed Shannon entropy from the normalized %RVC time series. Let ${x}_{i}(t)$ denote the %RVC amplitude of muscle $i\left(i=1,\dots ,10\right)$ at time point $t$. The normalized contribution of each muscle was defined as:

$${p}_{i}(t)=\frac{{x}_{i}(t)}{\sum_{j=1}^{10}{x}_{j}(t)+\varepsilon },$$

and Shannon entropy was calculated as:

$$H(t)=-\sum_{i=1}^{10}{p}_{i}(t){\mathrm{log}}_{2}{p}_{i}(t),$$

where $\varepsilon$ is a small constant to avoid division by zero. The theoretical maximum is ${H}_{\mathrm{max}}={\mathrm{log}}_{2}(10)\approx 3.32$, which occurs when all muscles contribute equally.

Entropy was computed sample-by-sample from the %RVC time series. For each task execution, an analysis window was defined from 0.5 s before the first keypress to 0.5 s after the last keypress, determined from the key-event logs. Within each execution, $H(t)$ was averaged across time points in the window to obtain a single entropy value for that execution. Each session contained eight task executions (two scores × two trials per score × two executions per trial; corresponding to the predefined task labels), and the session-wise entropy value was defined as the mean across the eight executions. This yielded a time series of up to 30 session-wise entropy values per participant.

For visualization and qualitative comparison of individual learning profiles, spaghetti plots were created for session-wise entropy. In addition, section-wise means (Sec1–Sec3; each comprising 10 sessions) were computed and used in descriptive scatter plots (with within-subject trajectories drawn from Sec1 to Sec3) to illustrate the relationships between task performance and entropy. No inferential statistics were performed for these descriptive scatter plots.

### Statistical analysis

To improve numerical stability and facilitate interpretation of the intercept, the session index was mean-centered within each analysis based on the observations included in the corresponding model (centered at the grand mean session).

#### Task performance (timing error)

Task performance was quantified as the mean absolute timing error (s). For each participant and each session, the outcome measure was computed as the mean across eight task executions, yielding one value per session (up to 30 sessions per participant). To model longitudinal change while accounting for repeated measures and inter-individual differences, we fitted a quadratic linear mixed-effects model (LMM) with subject-specific random effects:

$$\begin{aligned} {\mathrm{Error}}_{ij} &={\beta }_{0}+{\beta }_{1}\hspace{0.17em}{\mathrm{Session}\_\mathrm{c}}_{ij}+{\beta }_{2}\hspace{0.17em}{\mathrm{Session}\_\mathrm{c}}_{ij}^{2}\\ &\quad+{b}_{0i}+{b}_{1i}\hspace{0.17em}{\mathrm{Session}\_\mathrm{c}}_{ij}+{\varepsilon }_{ij},\end{aligned}$$

where ${\mathrm{Error}}_{ij}$ denotes the session-wise mean absolute timing error for subject $i$ at session $j$, and $\mathrm{Session}\_\mathrm{c}$ is the mean-centered global session index. For the task-performance dataset, $\mu =15.50$, such that $\mathrm{Session}\_\mathrm{c}=\mathrm{Session}-15.50$. $\beta$ terms are fixed effects, ${b}_{0i}$ and ${b}_{1i}$ are the subject-level random intercept and random slope for $\mathrm{Session}\_\mathrm{c}$, and ${\varepsilon }_{ij}$ is the residual error term. The model was specified in MATLAB (R2025b) as:

Error ~ 1 + Session_c + Session_c^2^ + (1 + Session_c | Subject).

Statistical significance of fixed effects was assessed using Satterthwaite’s approximation for degrees of freedom with a two-sided alpha level of 0.05. Model assumptions were evaluated using residual diagnostics (residuals vs fitted values, Q–Q plots, and residuals vs session).

#### Entropy: quadratic mixed-effects modeling

To examine longitudinal change in entropy across sessions while accounting for inter-individual differences, we fitted a quadratic LMM to the session-wise entropy values:

$$\begin{aligned} {\mathrm{Entropy}}_{ij} &={\beta }_{0}+{\beta }_{1}\hspace{0.17em}{\mathrm{Session}\_\mathrm{c}}_{ij}+{\beta }_{2}\hspace{0.17em}{\mathrm{Session}\_\mathrm{c}}_{ij}^{2}\\ &\quad+{b}_{0i}+{b}_{1i}\hspace{0.17em}{\mathrm{Session}\_\mathrm{c}}_{ij}+{\varepsilon }_{ij},\end{aligned}$$

where ${\mathrm{Entropy}}_{ij}$ denotes the session-wise entropy for subject $i$ at session $j$. $\mathrm{Session}\_\mathrm{c}$ is the session index centered at the grand mean of the observations included in the model. One observation (Sub10, Session 30) was excluded prior to model fitting because it was identified as a measurement failure during data acquisition; therefore, the centering constant for this dataset was $\mu =15.41$, such that $\mathrm{Session}\_\mathrm{c}=\mathrm{Session}-15.41$. Subject was included as a grouping factor with random intercepts and random slopes for $\mathrm{Session}\_\mathrm{c}$, allowing each participant to have an individual baseline level and linear trend component. The model was specified in MATLAB (R2025b) as:

$$\begin{aligned} \mathrm{Entropy} &\sim 1 + \mathrm{Session_c} + \: \mathrm{Session_c}^2 \\ & \quad + (1 + \mathrm{Session_c} \vert \mathrm{Subject}) \end{aligned}$$

Statistical significance of fixed effects was evaluated using Satterthwaite-approximated F tests ($\alpha =0.05$). Model assumptions were assessed using residual diagnostics (normal Q–Q plot, histogram of residuals, and residuals vs fitted values).

#### PCA-based description of coordination structure (loading-focused)

This pooled-PCA approach was adopted to define a single common reference axis for longitudinal comparisons across all sessions and participants; accordingly, learning-related change is evaluated primarily as score drift along this fixed axis. To describe multi-muscle coordination patterns, principal component analysis (PCA) was applied to the 10-muscle %RVC data as a descriptive linear decomposition of dominant covariation (loading) patterns. PCA outputs were not interpreted as direct evidence of coordination efficiency.

Data reduction to trial-level 10-dimensional vectors: For each task execution (trial), an analysis window was defined from 0.5 s before the first keypress to 0.5 s after the last keypress based on the key-event logs. Within this window, the %RVC time series of each muscle was time-averaged, yielding a single representative %RVC value per muscle. Thus, each execution was represented by a 10-dimensional vector (10 muscles × 1 time-averaged value), and each session provided eight such vectors (corresponding to the eight predefined task executions).

Pooled PCA across sections (single PC1 loading): To obtain a common descriptive axis of dominant covariation, PCA was performed once using all trial-level 10-dimensional vectors pooled across participants and across all sections (Sec1–Sec3) and sessions. The data matrix was constructed with task executions as observations and muscles as variables (10 columns). Each muscle variable was mean-centered across pooled observations prior to decomposition. The primary PCA outcome was the loading vector of the first principal component (PC1 loading; 10 elements), interpreted as the dominant covariation pattern (a muscle set describing which muscles tended to vary together). Because PCA loadings are sign-ambiguous, the sign of the pooled PC1 loading was fixed by enforcing a positive loading for the flexor digitorum superficialis (FDS).

PC1 score (projection onto the pooled PC1 axis): To obtain a scalar index of coordination for each subject and session on a common axis, each trial-level 10-dimensional vector was projected onto the pooled PC1 loading vector to compute a PC1 score. PC1 scores were then averaged across the eight task executions within each session, yielding one session-wise PC1 score per subject (up to 30 sessions). Longitudinal changes in PC1 scores were analyzed using linear mixed-effects models (LMMs) as described in the Results; the session index was mean-centered at the grand mean of the observations included in the PC1-score model (μ = 15.44).

## Results

### Task performance

Preliminary comparisons provided no clear evidence that score type (A vs B) materially affected timing accuracy or its session-dependent trend. Therefore, trials from the two scores were pooled for all subsequent analyses.

**Fig. 6:**
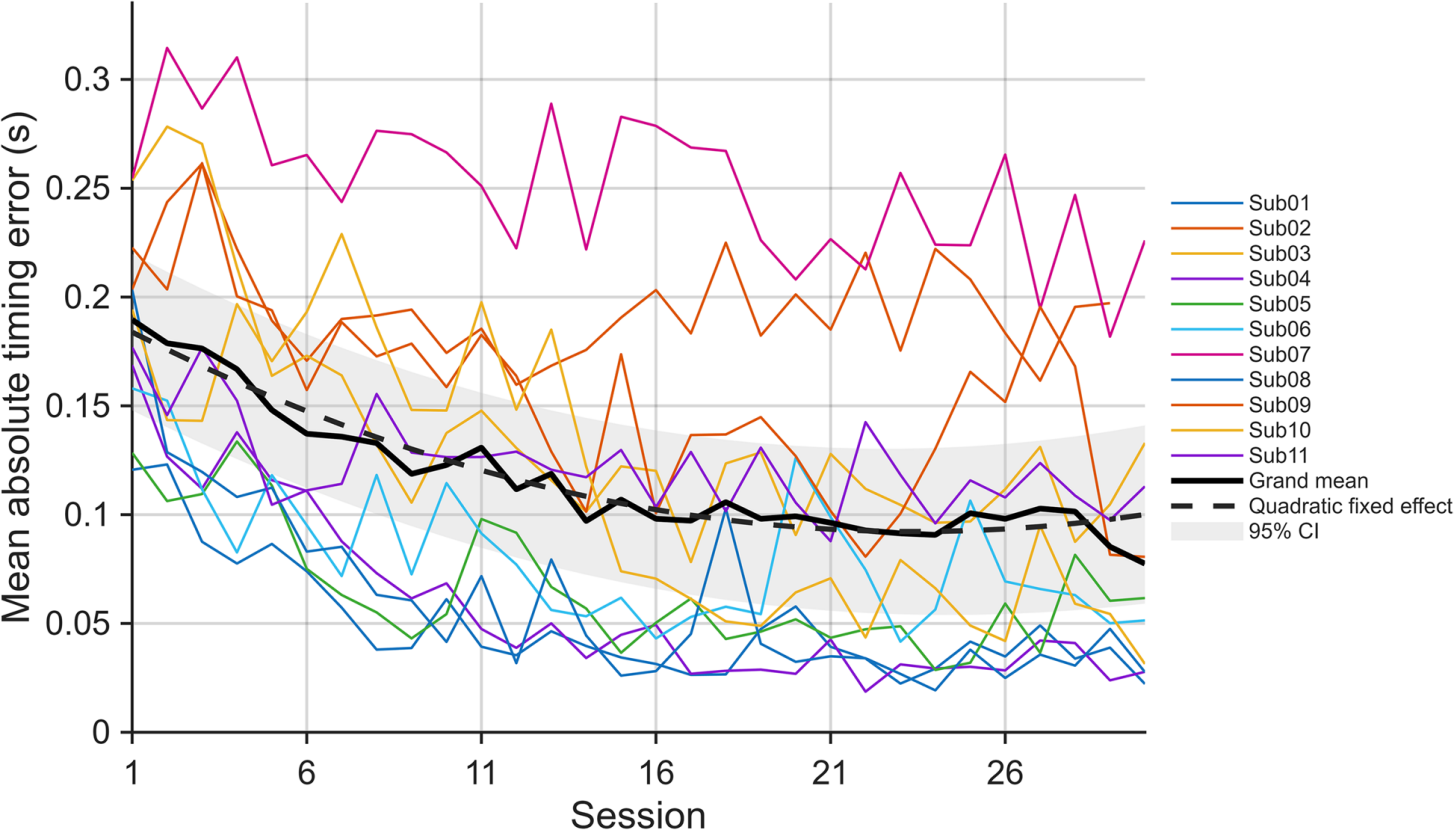
Task performance across 30 sessions

Across practice, mean absolute timing error decreased, showing a pattern consistent with relatively rapid early improvement followed by a gradual plateau (Fig. 6). This trend was supported by a quadratic linear mixed-effects model (LMM; Error ~ 1 + Session_c + Session_c^2^ + (1 + Session_c | Subject); ML estimation; 329 observations from 11 participants, up to 30 sessions), where Session_c denotes the session index centered at the grand mean for the task-performance dataset (μ = 15.50; Session_c = Session − 15.50). The fixed effect of Session_c was significantly negative (β = −2.91 × 10^−3^, SE = 4.26 × 10^−4^, *t*(326) = − 6.81, *p* = 4.57 × 10^− 11^), while the quadratic term was significantly positive (β = 1.82 × 10^ − 4^, SE = 1.96 × 10^− 5^, *t*(326) = 9.29, *p* = 2.23 × 10^− 18^), indicating diminishing returns in performance gains over sessions. The Satterthwaite-approximated ANOVA was consistent with these results, showing significant effects for both Session_c (F(1, 10.95) = 46.44, *p* = 2.97 × 10^− 5^) and Session_c^2^ (F(1, 307.06) = 86.34, *p* = 2.90 × 10^− 18^).

Random-effects estimates suggested substantial between-subject variability in baseline timing error (random-intercept SD = 0.0608) and individual linear trends (random-slope SD for Session_c = 0.00132), with a modest positive intercept–slope correlation (*r* = 0.232).

Model diagnostics did not indicate major violations of assumptions; residuals were approximately symmetric, with minor tail deviations and no clear session-dependent structure (Supplementary Fig. S2).

### Entropy

**Fig. 7:**
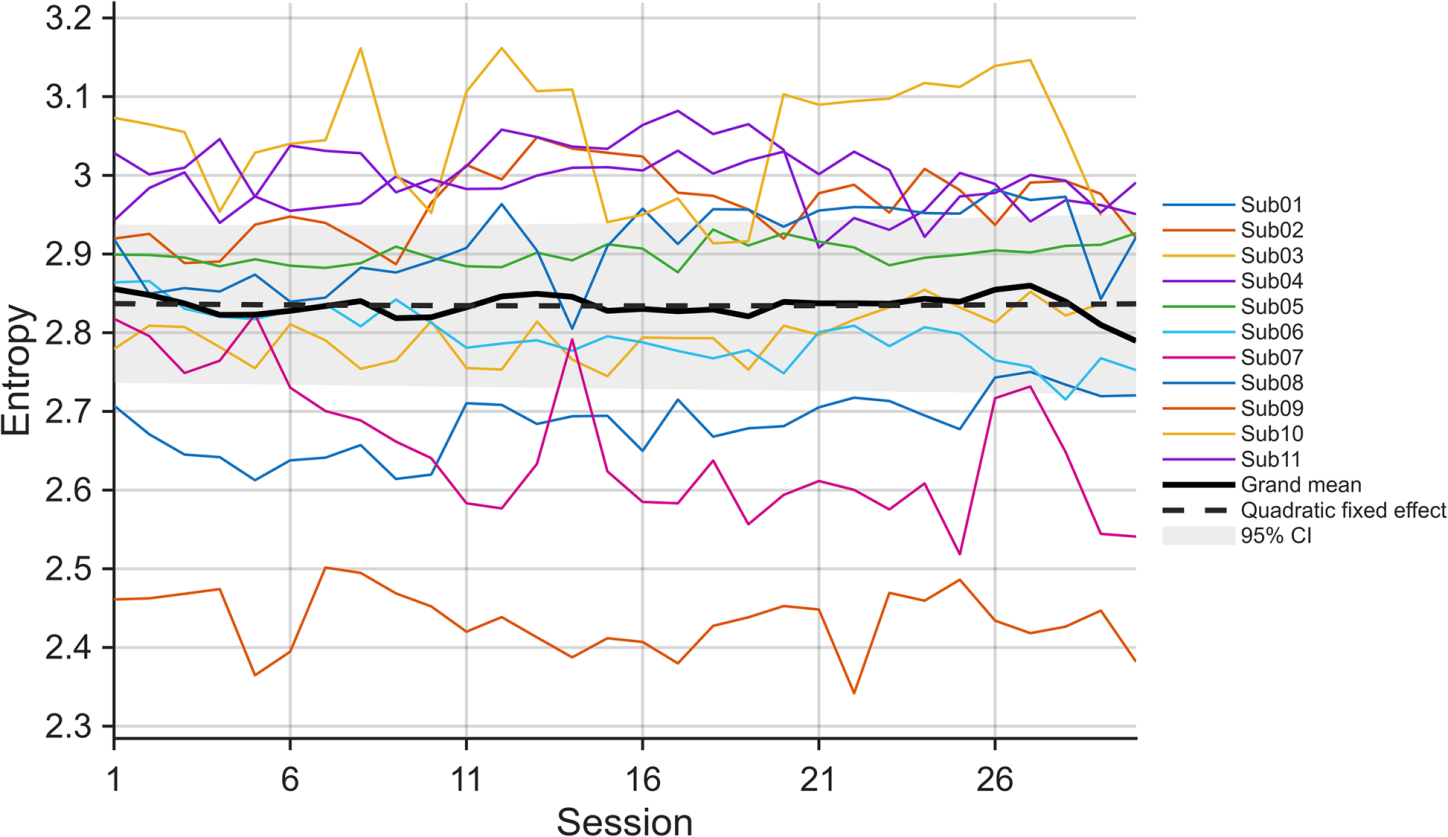
Time course of entropy across 30 sessions (all labels)

Entropy remained broadly stable across sessions at the group level, while pronounced between-subject differences persisted throughout practice (Fig. 7). A quadratic LMM (Entropy ~ 1 + Session_c + Session_c^2^ + (1 + Session_c | Subject); ML estimation; 328 session-level observations from 11 participants; entropy averaged within session) provided no evidence of a systematic session-dependent change. Here, Session_c was centered at the grand mean for the observations included in the entropy model (μ = 15.41; Session_c = Session − 15.41), reflecting the exclusion of one observation (Sub10, Session 30) due to a measurement failure during data acquisition.

Neither the linear term (Session_c: *β* = − 1.05 × 10^− 5^, SE = 8.38 × 10^− 4^, *t*(325) = − 0.013, *p* = 0.990) nor the quadratic term (Session_c^2^: *β* = 1.31 × 10^− 5^, SE = 3.61 × 10^− 5^, *t*(325) = 0.362, *p* = 0.718) was significant; the Satterthwaite ANOVA yielded consistent non-significant effects for Session_c (F(1, 11.03) = 0.00016, *p* = 0.990) and Session_c^2^ (F(1, 306.21) = 0.131, *p* = 0.718). In contrast, random-effects estimates indicated substantial inter-individual variability in baseline entropy (random-intercept SD = 0.1767) and variability in individual linear trends (random-slope SD for Session_c = 0.00262), consistent with heterogeneous subject-specific trajectories rather than a shared group-level time course.

Model diagnostics did not indicate major violations; residuals were approximately symmetric with mild tail deviations (Supplementary Fig. S3).

### EMG

**Fig. 8:**
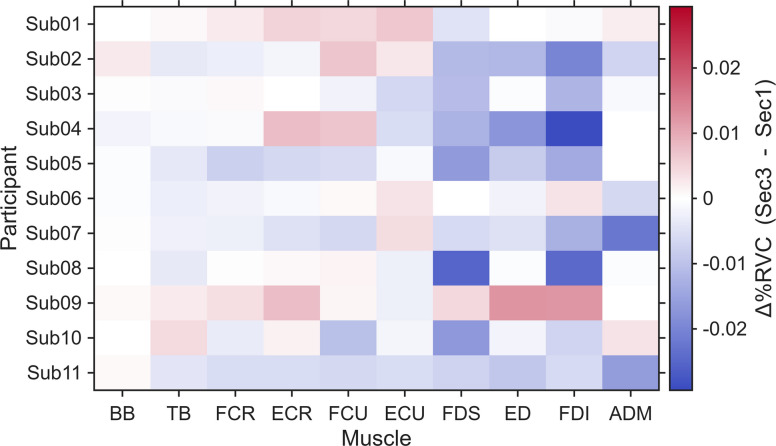
Changes in muscle activation amplitude between early and late learning (Δ%RVC, Sec3 − Sec1)

To provide a compact summary of learning-related changes in muscle activation amplitude, we computed the difference in RVC-normalized EMG amplitude between Sec3 and Sec1 for each participant and muscle (Δ%RVC = Sec3 − Sec1; Fig. 8). Overall, Sec3–Sec1 differences were small in magnitude and heterogeneous across participants and muscles, showing both positive and negative shifts depending on the individual. No consistent direction of change was apparent at the group level. These results suggest that improvements in task performance were not accompanied by a uniform, across-the-board increase or decrease in mean muscle activation amplitude, motivating subsequent analyses focused on coordination structure rather than amplitude alone.

**Fig. 9:**
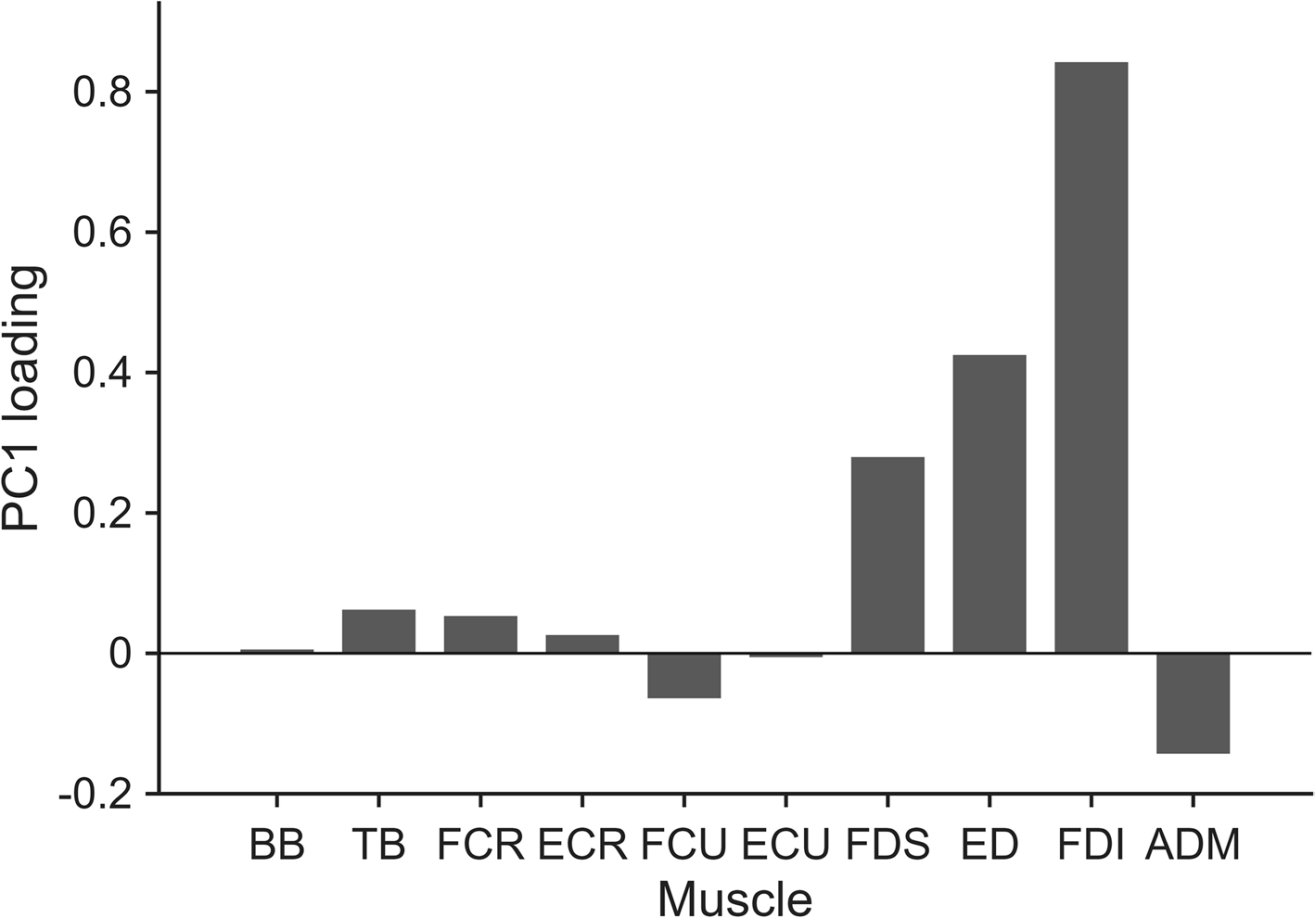
Pooled PCA across sections (Sec1–3): PC1 loading pattern (all labels)

To characterize coordination structure beyond mean amplitude, we performed a pooled PCA on the 10-muscle %RVC patterns using all trial-level vectors pooled across Sections 1–3 and participants to obtain a single dominant loading pattern (PC1 loading; Fig. 9). The sign of the pooled PC1 loading was fixed as described in the Methods (i.e., enforcing a positive loading for FDS). The resulting PC1 loading pattern indicated a dominant covariation structure characterized by a large positive contribution from FDI, additional positive contributions from ED and FDS, and a negative contribution from ADM, whereas the remaining muscles showed relatively small loadings.

**Fig. 10:**
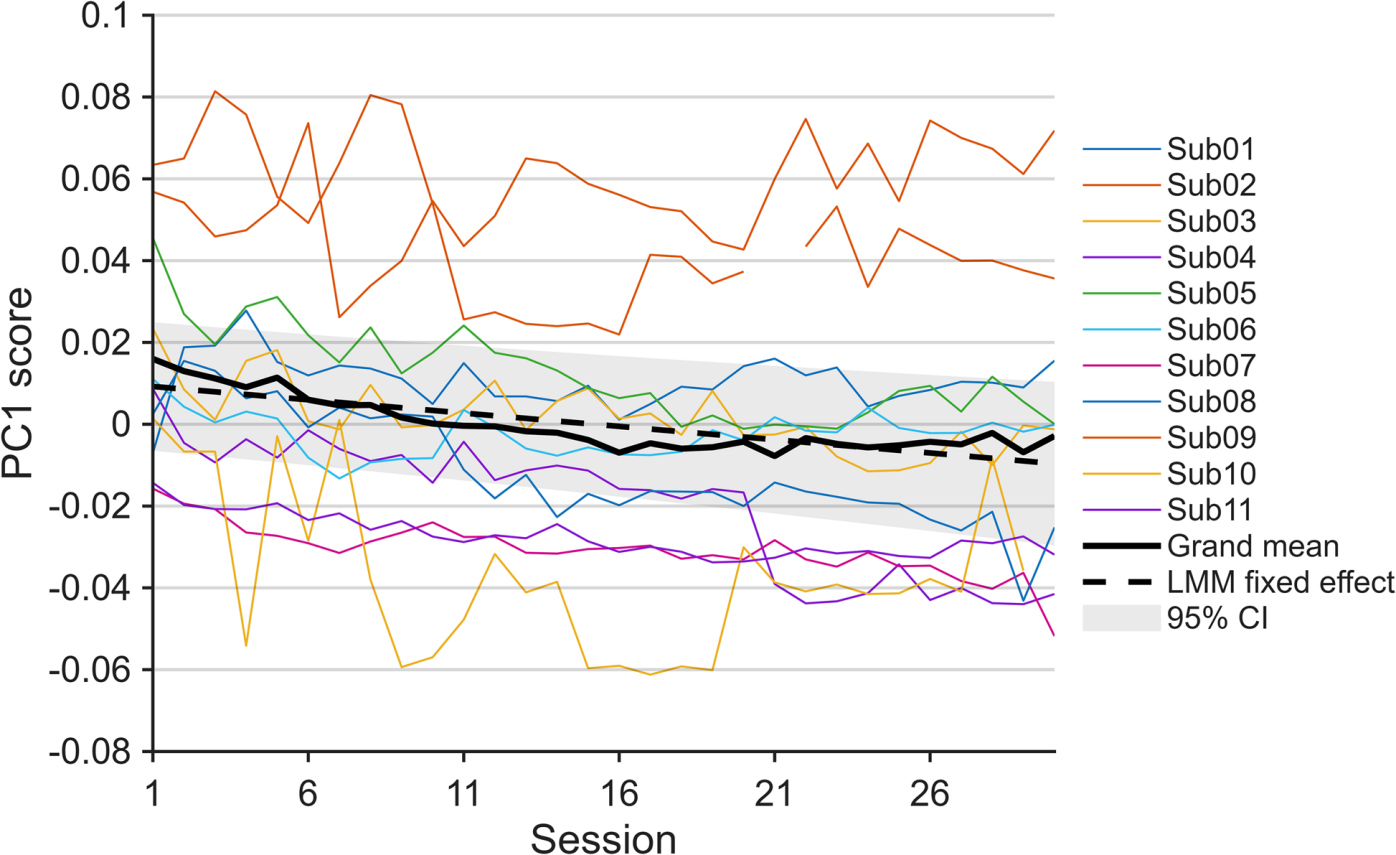
Trajectories of PC1 scores projected onto the common coordination axis (all labels)

Using the pooled PC1 loading axis as a common reference (Fig. 9), we quantified each participant’s coordination trajectory by projecting each trial-level 10-dimensional %RVC vector onto this axis and then averaging the resulting PC1 scores within each session (Fig. 10). At the group level, PC1 scores exhibited a modest negative drift across sessions, while between-subject offsets remained evident. A linear LMM (PC1score ~ 1 + Session_c + (1 + Session_c | Subject); session centered at the grand mean for the PC1-score model, *μ* = 15.44) indicated a significant fixed effect of Session_c (*β* = − 6.50 × 10^− 4^, SE = 1.95 × 10− 4, *t*(11.02) = − 3.34, *p* = 6.62 × 10^− 3^; Satterthwaite ANOVA: F(1, 11.02) = 11.13, *p* = 6.62 × 10^− 3^). The fixed intercept was not significantly different from zero (*β* = − 1.54 × 10− 4, *p* = 0.986), indicating that the estimated mean PC1 score at the centered session was close to zero. Overall, these results suggest gradual shifts along a common pooled coordination axis, rather than implying a section-specific reorganization of the dominant loading pattern.

Diagnostic plots suggested an acceptable fit, with approximately symmetric residuals and mild tail deviations (Supplementary Fig. S4).

Fixed-effect estimates for the mixed-effects models used in Figs. 6, 7, and 10 are summarized in Table 1 (estimates, SEs, df, *p*-values, and 95% CIs).

**Table 1:**
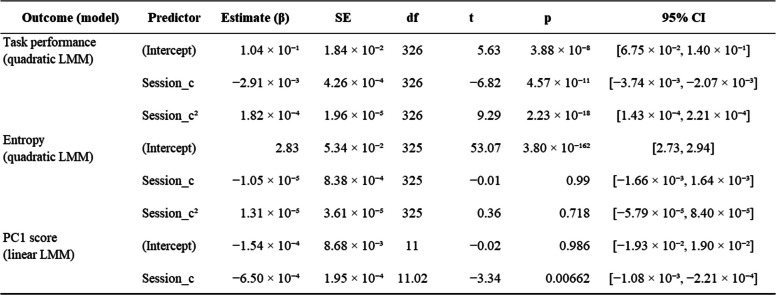
Fixed effects from linear mixed-effects models of task performance, entropy, and PC1 score

Linear mixed-effects models were fitted by maximum likelihood. Subject was included as a grouping factor with random intercepts and random slopes for Session_c ((1 + Session_c | Subject)). Session_c denotes the session index mean-centered within each dataset; Session_c^2^ indicates the squared centered session term. For task performance, the dependent variable was session-wise mean absolute timing error (s). For entropy, values represent session-wise entropy averaged across labels (all labels). PC1 scores were computed by projecting each session’s %RVC muscle-activation pattern onto the common PC1 loading axis (sign-aligned as described in Fig. 9). Degrees of freedom and *p*-values for fixed effects were obtained using the Satterthwaite approximation. *CI* Confidence interval, *SE* Standard error

**Fig. 11:**
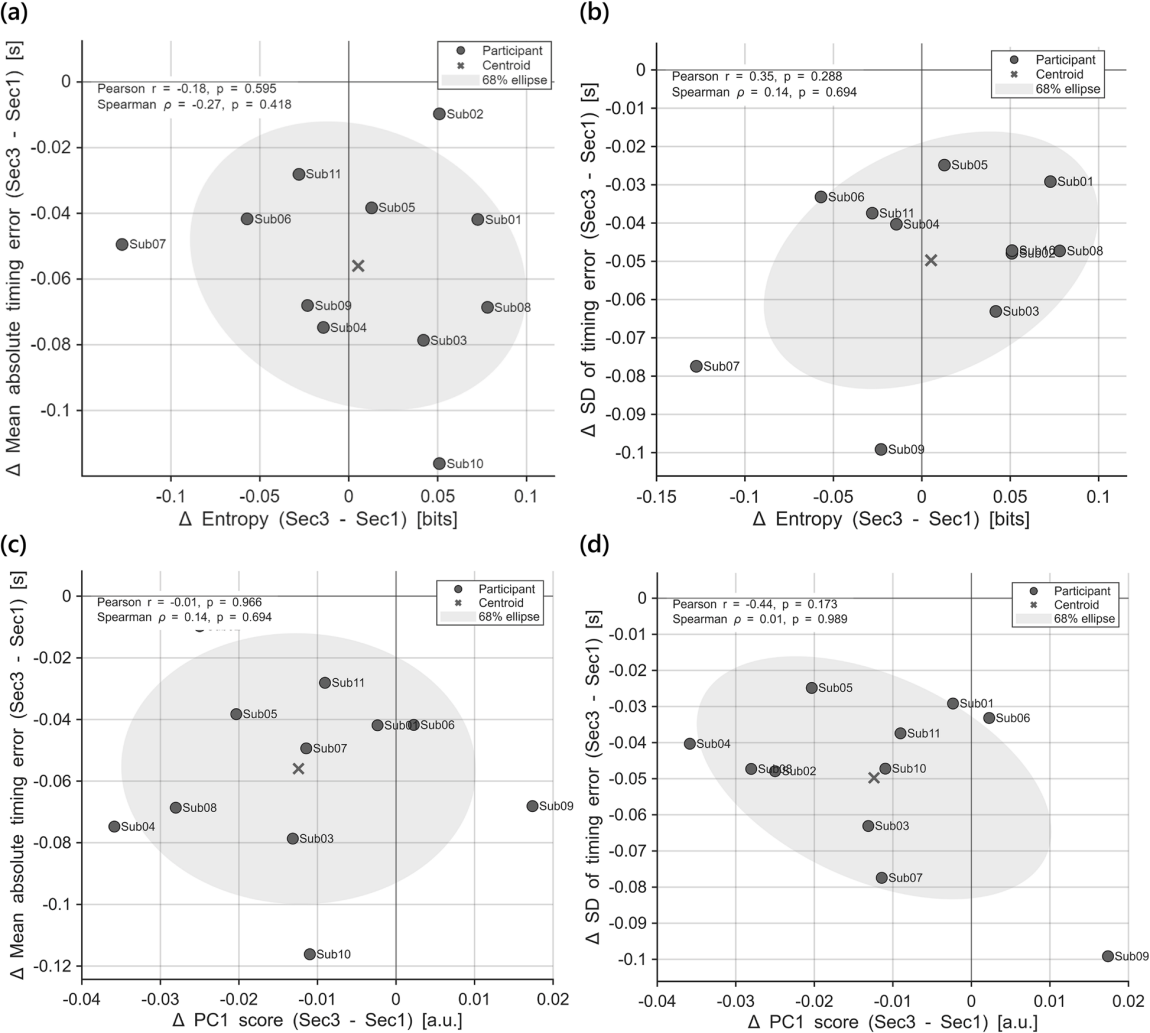
Associations among learning-related changes in performance, variability, entropy, and PC1 score

To explore cross-metric associations in learning-related changes, we computed change scores between late and early practice for each participant (Δ = Sec3 − Sec1) and examined their pairwise relationships (Fig. 11; *n* = 11). Changes in entropy were not significantly associated with improvements in mean absolute timing error (Pearson *r* = − 0.18, *p* = 0.595; Spearman *ρ* = − 0.27, *p* = 0.418; Fig. 11a) or with changes in within-session performance variability (timing-error SD; *r* = 0.35, *p* = 0.288; *ρ* = 0.14, *p* = 0.694; Fig. 11b). Likewise, changes in PC1 score were not significantly associated with changes in mean absolute timing error (Pearson *r* = − 0.01, *p* = 0.966; Spearman *ρ* = 0.14, *p* = 0.694; Fig. 11c) or timing-error SD (Pearson *r* = − 0.44, *p* = 0.173; Spearman *ρ* = 0.01, *p* = 0.989; Fig. 11d). Given the small sample size (*n* = 11) and the exploratory nature of these analyses, these correlations should be interpreted cautiously.

## Discussion

Across 30 practice sessions of a fixed, tempo-constrained finger sequence task, we observed four principal findings. First, task performance improved robustly over practice and was best characterized by a decelerating (quadratic) learning curve, with the largest reduction in timing error occurring in the early phase and a gradual plateau thereafter. Second, the group-level trajectory of EMG entropy did not show a consistent monotonic trend across sessions; however, there was substantial inter-individual heterogeneity, indicating that both baseline entropy and its session-to-session change were strongly participant dependent. Third, changes in normalized EMG amplitude (Δ%RVC) from the early to late phase were small in magnitude and non-uniform across muscles and participants, which is not consistent with a single global mean EMG amplitude increase/decrease account of learning-related changes. Finally, when multi-muscle activity was represented on a common PCA-derived axis defined from pooled trial-level %RVC patterns, the corresponding PC1 scores showed a modest but systematic drift across sessions. This pattern indicates a gradual shift in the position of the activation patterns along a fixed, dataset-level covariation dimension, without implying section-specific redefinition of the dominant loading structure.

Motor learning is commonly defined as a set of processes associated with practice or experience that leads to relatively permanent changes in the capability for skilled performance. In this context, the quadratic improvement in timing accuracy observed here is consistent with a canonical, negatively accelerated learning curve in which rapid early gains are followed by diminishing returns as performance approaches a task- and constraint-specific asymptote [1]. In the present paradigm, participants repeatedly practiced the same fixed sequence under stable task constraints (e.g., prescribed tempo, invariant key–lane mapping, and consistent feedback rules), a practice structure that is generally sufficient to produce measurable improvements in performance over time when the goal is a reproducible skill under repeated conditions [17]. The early phase may reflect relatively fast improvements in task-specific timing and stimulus–response mapping, whereas later sessions may be dominated by fine-tuning processes that yield smaller incremental benefits [1]. However, the present dataset does not allow us to attribute the behavioral gains to a specific mechanism (prediction, planning, or execution refinement). Accordingly, the emergence of an apparent plateau can be interpreted as (i) a ceiling imposed by fixed temporal constraints and measurement resolution, (ii) a shift in the speed–accuracy balance once basic task requirements are met, and/or (iii) saturation of the most readily accessible gains within the practiced schedule. Importantly, because task conditions were held constant across sessions, the behavioral learning curve provides a useful reference point for interpreting neuromuscular indices: performance can improve substantially even when group-average EMG-derived measures do not converge toward a single trajectory, motivating a discussion centered on individualized routes to skill acquisition under identical task demands. At the same time, EMG-derived summaries are descriptive and do not uniquely identify underlying control mechanisms.

Despite clear behavioral learning, EMG entropy did not exhibit a uniform group-level change across sessions. This null fixed-effect finding is informative rather than inconclusive, because it indicates that the acquisition of timing accuracy under fixed task constraints does not necessarily require a stereotyped, monotonic shift in the spatial distribution of muscle activation captured by entropy. Here, entropy is interpreted as a compact summary of the evenness (dispersion) of an activation-amplitude distribution across channels, grounded in information theory [25, 26]; when applied to spatial EMG, entropy is maximized when RMS amplitudes are equal across electrodes and decreases as activity becomes more localized [35]. In other words, improvements in performance can be achieved without converging toward a single group-average pattern of “more/less complex” muscle activation. This dissociation cautions against interpreting entropy as a universal proxy for learning progress and instead supports the view that entropy reflects one descriptive aspect of neuromuscular state that may remain stable, fluctuate, or shift idiosyncratically depending on how each individual organizes muscle activity to meet the task demands. More broadly, given the redundancy of the motor system, multiple neuromuscular solutions can achieve similar task outcomes [10, 11], which makes the absence of a single group-average entropy trajectory plausible.

At the same time, the mixed-model results suggested substantial between-subject variability in both baseline entropy and its session-related change, highlighting that individual trajectories constitute the primary signal in this dataset. Such heterogeneity implies that the observed data are consistent with multiple distributional/coordination regimes (relatively stable activation distributions alongside improving timing, versus gradual redistribution across channels), without these strategies producing a coherent group-average trend. This emphasis on person-specific time courses is consistent with contemporary views that motor learning is individualized and that trajectories can differ markedly across individuals even under the same practice schedule [12]. Importantly, while entropy is often discussed in relation to variability, flexibility, or exploration, the present results do not justify treating entropy as a direct index of “exploration” or “optimality.” Rather, in the context of multi-muscle EMG, entropy should be interpreted conservatively as a compact summary of the distributional spread of activation across channels at a given time point or epoch, and its functional meaning depends on task constraints and accompanying measures [30]. Entropy-based measures have also been applied to EMG to characterize state-related changes (e.g., fatigue), supporting their utility as descriptive markers while reinforcing the need for context-aware interpretation [27–29]. Establishing a mechanistic link between entropy changes and specific control processes would require additional information (kinematics/kinetics, task constraint manipulations, or analyses that partition variability into task-relevant and task-irrelevant components), which we address as future directions.

Changes in normalized EMG amplitude from the early to late phase (Δ%RVC) were generally small and did not show a consistent direction across muscles or participants. This pattern argues against a simple learning account in which performance improvements are primarily driven by global increases (or decreases) in EMG amplitude (%RVC). Instead, the data suggest that, within this task and measurement context, learning-related performance gains can occur with minimal net change in overall activation level at the group level. Small and heterogeneous Δ%RVC also implies that amplitude-based summaries alone may be insufficient to capture the relevant adaptations, because they conflate multiple processes (changes in co-contraction, redistribution across synergists/antagonists, or trial-to-trial modulation) into a single scalar quantity. This interpretation is consistent with a systematic review showing that changes in motor performance are only weakly related to changes in quantitative EMG measures such as amplitude and duration, and that understanding practice-related changes often requires additional information on timing characteristics, co-activation, and kinematic/kinetic variables [36]. Accordingly, the absence of a uniform amplitude shift motivates analyses that focus on how activation is distributed and coordinated across muscles, rather than how much activation is produced overall.

The pooled PCA identified a dominant covariation pattern across the entire dataset, which we used as a common descriptive reference axis. Because the axis was defined from pooled observations, the PC1 loading should be interpreted as a dataset-level summary of the dominant covariance structure rather than evidence that loading structure remained unchanged across sections. Learning-related changes were therefore assessed primarily as shifts in PC1 scores (projections) along this fixed axis. Here, “coordination structure” refers to functional patterns of relationships among muscles—how roles are distributed and how muscles covary during task execution [16]. Importantly, in this context PCA is used as a descriptive decomposition of multi-muscle activity into low-dimensional covariation patterns, not as a direct measure of efficiency, optimality, or neural modules. In the present pooled-PCA framework, the PC1 loading is fixed by construction and thus does not, by itself, test whether the underlying covariance structure is invariant across sections. Nevertheless, the fact that learning-related change was captured primarily as score drift along this common axis is compatible with the interpretation that adaptation may be expressed as changes in the expression of dominant covariation patterns (scores) or subtler redistribution across dimensions, rather than a wholesale replacement of the dominant axis. This interpretation is consistent with modular accounts of motor control in which movements can be generated through combinations of a limited set of muscle synergies and adaptation can be expressed through changes in synergy recruitment (weighting) rather than wholesale changes in synergy structure.

While the dominant covariation axis defined by the pooled PCA was used as a fixed common reference, PC1 scores exhibited a modest but systematic session-related drift. This combination—stable axis with changing projection—suggests that learning could have proceeded through gradual changes in how strongly the existing dominant coordination pattern was expressed, rather than through a redefinition of the pattern itself. One parsimonious interpretation is that the session-related drift may be compatible with gradual changes in the balance of activation patterns along the dominant covariation dimension; however, the present data do not allow attribution to a specific control mechanism. Such a “stable structure with changing expression/weighting” pattern is consistent with reports from adaptation paradigms in which baseline synergy structure can remain similar while performance is restored through altered recruitment patterns [37], and more broadly with perspectives that motor learning can involve multiple strategies including modulation of co-contraction or redistribution/alternation among muscles depending on task demands [21, 23]. Importantly, because the present dataset does not include kinematic/kinetic measures or independent markers of co-contraction and endpoint mechanics, we refrain from attributing this drift to specific control mechanisms (increased efficiency or reduced unnecessary activation). Instead, the key implication is descriptive: under identical task constraints, the principal coordination structure can remain stable while the state along that structure shifts over practice, providing an interpretable intermediate level between raw EMG amplitude and overt performance.

Across participants, improvements in performance were not strongly or consistently coupled to changes in entropy or coordination scores when summarized as early-to-late differences, indicating that the mapping between behavioral gains and neuromuscular indices is not well described by a simple monotonic relationship. This weak cross-metric coupling is consistent with at least three non-exclusive possibilities. First, the metrics capture partially distinct facets of the neuromuscular state (distributional spread, covariation structure, and low-dimensional expression) that need not change in parallel with timing error. Second, individuals may reach similar behavioral outcomes via different neuromuscular routes, yielding cancellation at the group level and attenuated across-subject associations. Third, the timing of changes may be misaligned across measures (early performance gains preceding slower coordination drift), so that a single early–late contrast obscures temporally staggered adaptations. In addition, given the modest sample size and the multiplicity of tested associations, these cross-metric relationships should be regarded as exploratory. Collectively, these findings reinforce the rationale for focusing on longitudinal, participant-specific trajectories rather than expecting a one-to-one correspondence between any single EMG-derived summary and behavioral learning, in line with contemporary perspectives emphasizing individualized learning dynamics [12].

Our observation of heterogeneous EMG-derived trajectories under identical task constraints is consistent with the view that learners may adopt different search routes, although the present data cannot specify the underlying control variables without additional kinematic/kinetic measures [13, 14]. Taken together, the present findings extend prior work on inter-individual variability in motor learning by showing how such variability manifests across complementary levels of description under identical task constraints. Rather than treating “individual differences” as residual noise around a group mean, we explicitly separated (i) trajectory-level differences in longitudinal change (e.g., learning curve shape, participant-specific entropy trajectories, and score drift) from (ii) structure-level properties of multi-muscle coordination (e.g., preservation of a dominant covariance axis). This decomposition provides a practical framework for describing “different routes to similar outcomes”: participants can achieve comparable behavioral improvements while showing stable entropy, drifting PC1 scores, or minimal amplitude change, because the task can be solved through multiple neuromuscular organizations that do not necessarily converge toward a single canonical pattern. In this sense, the contribution of the study is not to propose a new mechanistic model of control, but to offer an empirically grounded, longitudinal characterization strategy that bridges performance, distributional summaries, and coordination structure in multi-channel forearm EMG.

Several limitations should be acknowledged when interpreting these results. First, we did not record detailed kinematic or kinetic variables (e.g., finger trajectories, joint angles, endpoint forces), which constrains mechanistic inference: changes in entropy or PC scores cannot be uniquely mapped onto specific movement strategies, co-contraction, or reductions in task-irrelevant variability. This limitation is particularly important because practice-related performance changes are not necessarily well explained by quantitative EMG measures alone, and prior work has emphasized the value of combining EMG with movement and force measurements to interpret learning-related adaptations [36]. Second, the practiced sequence and task constraints were fixed across sessions, which likely encouraged anticipatory timing and sequence-specific learning; thus, improvements in timing error may reflect a mixture of motor execution refinement and predictive/sequence learning, and the relative contribution of these components cannot be disentangled in the current design. Third, the sample size was modest and the task was specific, limiting the generalizability of the identified patterns across populations, effectors, and task families. Finally, entropy and PCA-derived metrics are descriptive summaries of multi-muscle EMG; while they are useful for compactly characterizing distributional spread and covariance structure, they do not by themselves determine whether observed variability is “good” or “bad,” nor do they establish causality with respect to performance improvements. These limitations motivate targeted extensions in future work rather than undermining the central descriptive conclusions of the present study.

Future work can strengthen the interpretability of entropy- and PCA-based EMG summaries by integrating complementary measurements and experimental manipulations. A first priority is concurrent kinematic and kinetic recording (e.g., finger motion, joint angles, and keypress/endpoint forces), which would allow coordination drift and entropy changes to be linked to concrete movement strategies such as altered finger independence, joint-level synergies, and co-contraction. Second, to move beyond descriptive variability, analyses that partition variability into task-relevant and task-irrelevant components (e.g., uncontrolled manifold–type approaches or related structure–function decompositions) could clarify whether participant-specific entropy trajectories correspond to beneficial stabilization, compensatory strategies, or merely redistribution without functional consequence. Third, systematic manipulation of task constraints (tempo, accuracy demands, sensory feedback, or sequence uncertainty) would test the sensitivity and specificity of entropy and coordination metrics: if these indices truly reflect adaptive reorganization, their trajectories should change predictably as constraints shift. Finally, an important extension will be to connect the observed individual trajectories to determinants of learning at the individual level (neural or sensory factors), consistent with evidence linking inter-individual differences in motor learning to brain structure/function and proprioceptive characteristics [15, 16]. Together, these extensions would enable a more mechanistic interpretation of how individuals traverse different neuromuscular routes while learning under shared behavioral goals.

## Conclusion

In conclusion, participants demonstrated robust behavioral learning characterized by a decelerating improvement in timing accuracy across practice. In contrast, EMG-derived indices did not converge toward a single group-average neuromuscular trajectory: entropy showed substantial individual heterogeneity, amplitude changes were small and non-uniform, and the dominant coordination axis was preserved while its expression drifted modestly over time. These results underscore that meaningful performance gains can emerge through diverse, participant-specific neuromuscular organizations, and they support a longitudinal approach that jointly considers trajectories and coordination structure when characterizing individual differences in motor learning.

## Supplementary Information

Supplementary Material 1: Figure S1-S5.

## Data Availability

No datasets were generated or analysed during the current study.
