# Supplementary material for: Longitudinal analysis of neuromuscular adaptation using entropy and PCA during motor learning
Source: J Physiol Anthropol. 2026 May 15;45:16. doi: 10.1186/s40101-026-00432-y (PMC13348549; doi:10.1186/s40101-026-00432-y)
Supplement: Supplementary file 1 — Supplementary Material 1: Figure S1-S5. [file 40101_2026_432_MOESM1_ESM.docx]

# Supplement


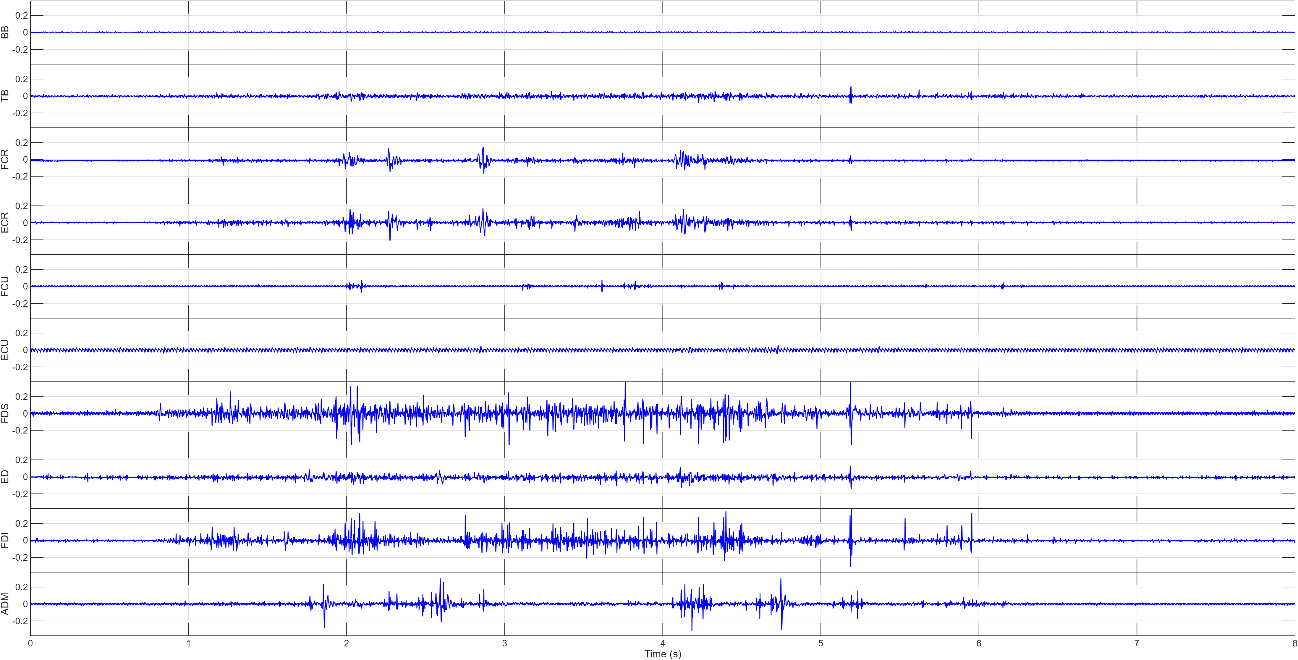


## Supplementary Figure S1. Representative raw sEMG signals from a single trial

From top to bottom: Biceps brachii (BB), Triceps brachii (TB), Flexor carpi radialis (FCR), Extensor carpi radialis (ECR), Flexor carpi ulnaris (FCU), Extensor carpi ulnaris (ECU), Flexor digitorum superficialis (FDS), Extensor digitorum (ED), First dorsal interosseous (FDI), and Abductor digiti minimi (ADM).
Signals are shown as raw waveforms before filtering to illustrate baseline stability and task-related burst activity. The horizontal axis represents time (s), and the vertical axis represents voltage (gain = 2000).


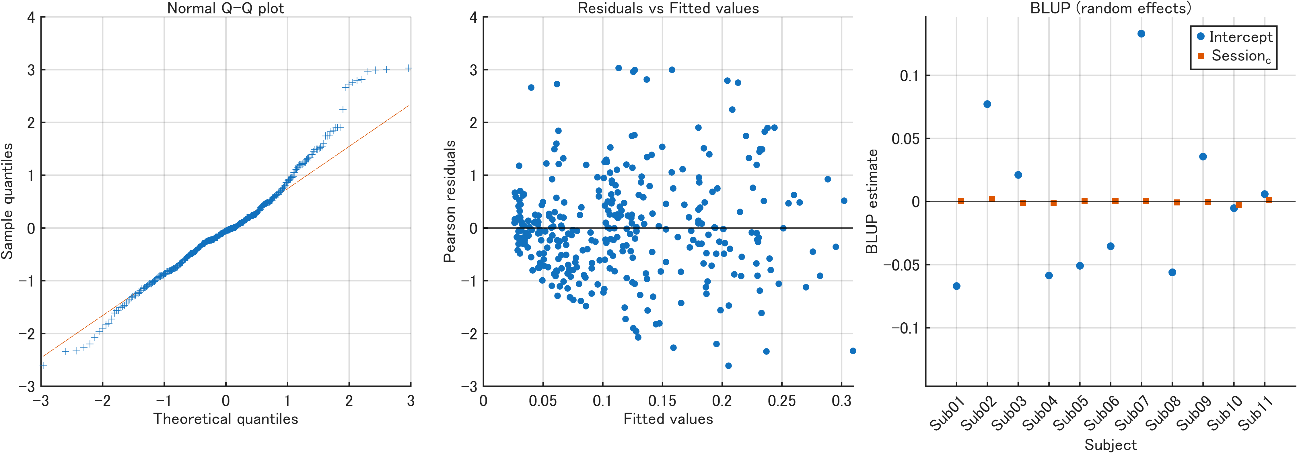


## **Supplementary Figure S2. Model diagnostics for the quadratic mixed-effects model in Fig. 6**

Normal Q–Q plot of Pearson residuals, residuals versus fitted values, and best linear unbiased predictions (BLUPs) of subject-specific random effects (intercept and slope). Overall, the diagnostics were broadly consistent with an adequate model fit. Minor deviations from normality were observed in the upper tail, and visual inspection did not reveal an obvious systematic pattern in residuals with respect to fitted values.


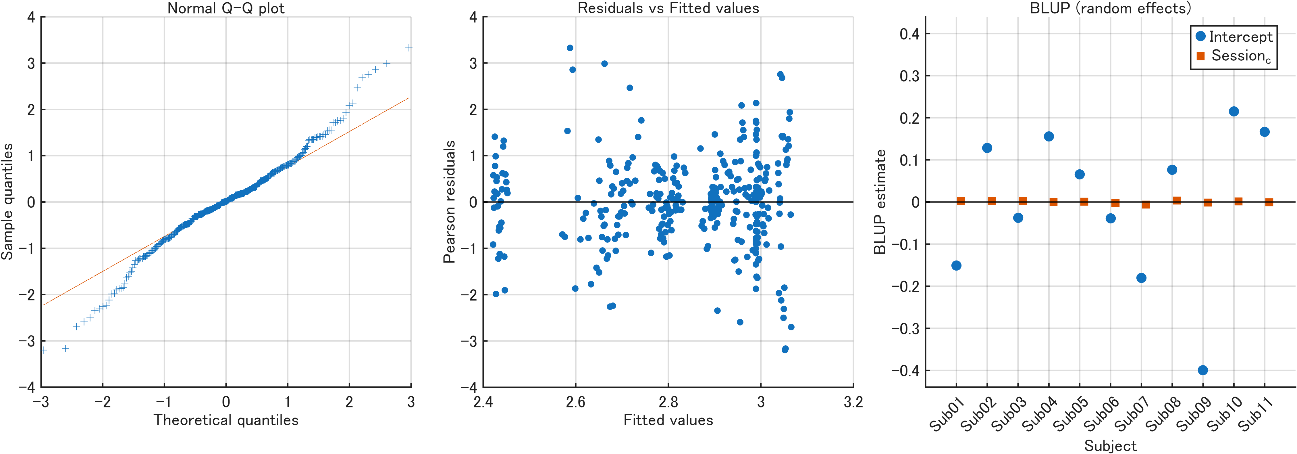


## **Supplementary Figure S3. Model diagnostics for the quadratic mixed-effects model in Fig. 7**

Normal Q–Q plot of Pearson residuals, residuals versus fitted values, and BLUPs of subject-specific random effects (intercept and slope). The diagnostics were broadly consistent with an adequate fit. Modest deviations from normality were observed in the tails, and there was no strong visual indication of pronounced heteroscedasticity or structured residual patterns across fitted values.


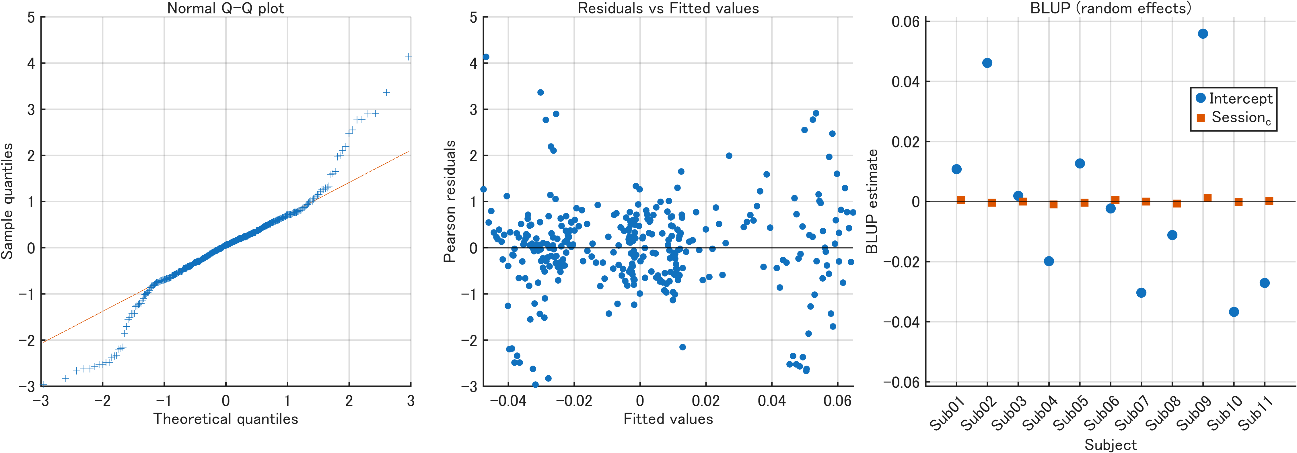


## **Supplementary Figure S4. Model diagnostics for the PC1score mixed-effects model in Fig. 10**

Normal Q–Q plot of Pearson residuals, residuals versus fitted values, and BLUPs of subject-specific random effects (intercept and slope). The diagnostics were broadly consistent with an adequate fit, with mild tail deviations from normality. Residuals did not show an obvious systematic pattern with respect to fitted values based on visual inspection.


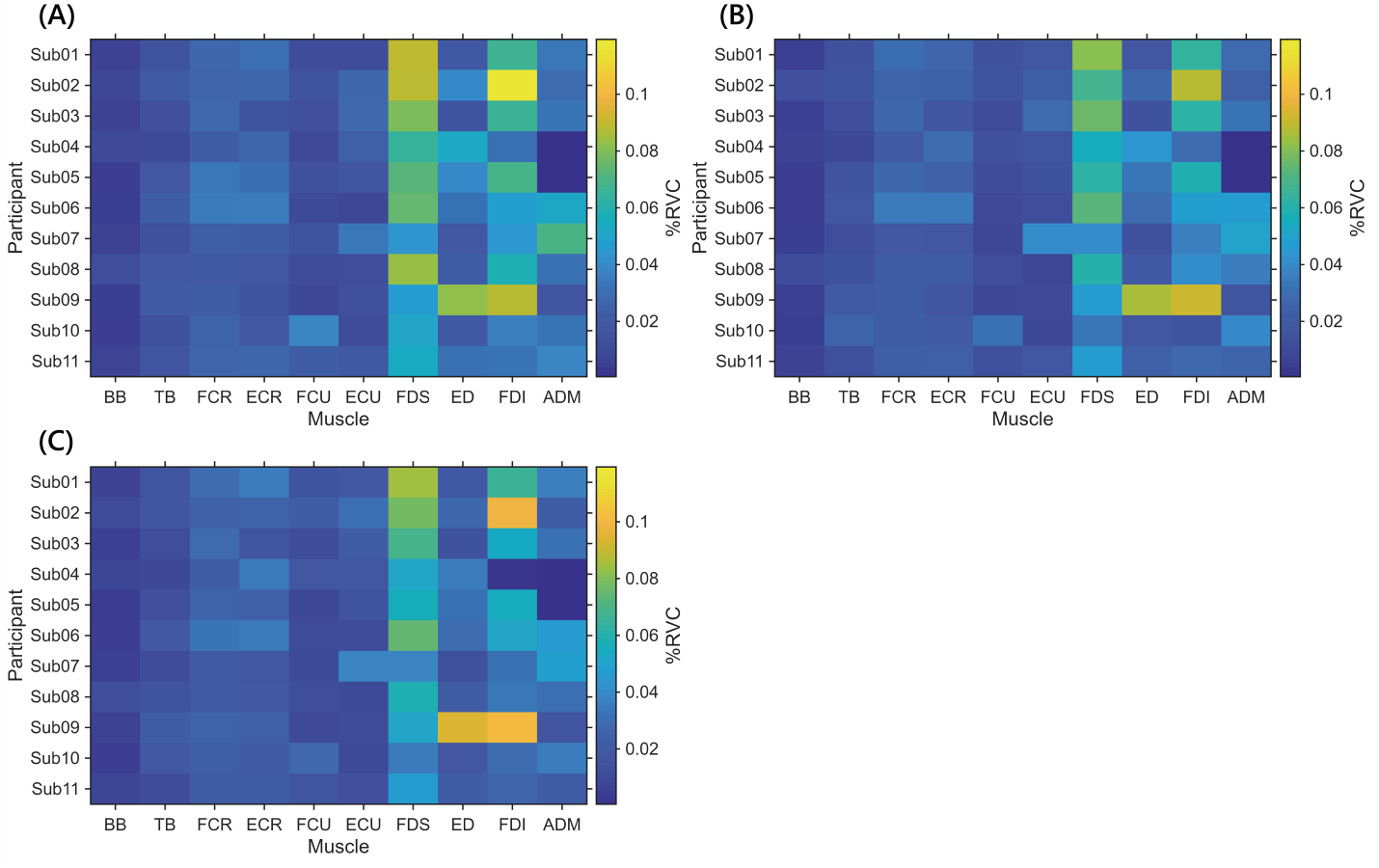


## Supplementary **Figure S5. %RVC heatmaps (Subject × Muscle) across sections**

**Heatmaps of normalized EMG amplitude (%RVC) summarized for each participant (rows: Sub01–Sub11) and each recorded muscle (columns) for (A) Section 1, (B) Section 2, and (C) Section 3. Each cell represents the participant-wise mean %RVC value computed from RMS EMG and averaged across all trials/sessions included in the corresponding section. Warmer colors indicate higher normalized activation. The same color scale is used across panels to facilitate comparison of activation patterns between sections, muscles, and participants.**
